# Open circuit fault localization in dual active bridge based simultaneous battery charging systems using multi label classification

**DOI:** 10.1038/s41598-026-52101-w

**Published:** 2026-05-18

**Authors:** Khaled Sayed Abd El-Naeem, Mohamed A. Nayel, Mohamed Abdelrahem, Islam Alkabbany

**Affiliations:** 1 Electrical Engineering Department, Assiut University, Assiut, 71515 Egypt; 2 Chair of High-Power Converter Systems, Technical University of Munich, 80333 Munich, Germany

**Keywords:** Isolated dc-dc converters, Multi-label fault diagnosis, Deep learning, Time-frequency analysis, Energy science and technology, Engineering, Mathematics and computing

## Abstract

This paper presents a multi-label fault localization framework for open-circuit fault diagnosis in a three-port Dual Active Bridge (DAB) converter using deep learning. The proposed method leverages time-frequency features extracted from midpoint voltage signals through the continuous wavelet transform (CWT) to generate multi-channel scalogram images. These images are then used to train a ResNet-18 convolutional neural network (CNN) for simultaneous detection and localization of single and multiple switch faults. The dataset was generated under various state-of-charge (SOC) and fault timing conditions to ensure comprehensive coverage of the converter. Simulation results demonstrate high diagnostic accuracy, with both micro- and macro-F₁ scores exceeding 99% on unseen test data. Compared to an equivalent multi-class ResNet-18 classifier, the proposed multi-label network achieved approximately 3% higher macro-F₁ score and better generalization to simultaneous fault conditions. Moreover, the model generalizes effectively to three-switch faults, achieving a micro-F₁ score of approximately 85% despite being trained only on single- and two-switch cases. Robustness analyses further confirm stable performance under dead-time variations, measurement noise, and sensor reduction, maintaining over 92% F₁-score accuracy with only two voltage sensors. In addition, real-time performance evaluation shows that the proposed framework achieves an online diagnosis latency of 136.8 ms. These findings highlight the model’s effectiveness for fault diagnosis in multi-port power converters.

## Introduction

The rapid growth of electric vehicles (EVs) has created a significant demand for efficient and reliable charging infrastructures. As the number of EVs increases, conventional single-port chargers are no longer sufficient to meet large-scale charging requirements. Therefore, multi-port and simultaneous charging systems have emerged as a promising solution, enabling multiple batteries or vehicles to be charged simultaneously from a common DC link, reducing installation costs, and improving system utilization efficiency^1,2^.

In such simultaneous charging systems, the DAB converter has gained wide adoption due to its high-power density, galvanic isolation, bidirectional power flow, and soft-switching capability. These characteristics make DAB converters highly suitable for multi-port energy conversion systems, where multiple secondary ports share a single high-frequency transformer network for power transfer among multiple batteries or energy storage units^2,3^.

Despite these advantages, the reliability of DAB-based systems remains a major concern. Power semiconductor switches are susceptible to various types of faults, particularly open-circuit (OC) and short-circuit (SC) faults^4–6^, which can lead to power imbalance, increased current stress, distorted waveforms, and even catastrophic converter failure^7,8^. SC faults are associated with extremely fast current rise and high destructive energy levels, necessitating immediate protection mechanisms at the hardware level. Although open-circuit faults do not usually interrupt converter operation from working immediately, prolonged operation under these conditions may increase the stress on healthy devices, which may lead to secondary faults^9^.

In addition to these fault mechanisms, practical switches also exhibit non-ideal behaviors such as rising and falling times. To prevent shoot-through during switching transitions, a mandatory dead time must be inserted between the turn-off of one switch and the turn-on of its complementary device. This dead time constitutes a significant form of control-signal delay, intentionally introduced to ensure safe operation, yet it inevitably shifts the effective switching instants and alters midpoint voltage transitions^10^. As a result, the transient features used for fault diagnosis may be distorted.

In multi-port charging applications, semiconductor faults can propagate through the magnetically coupled DAB structure, affecting multiple ports simultaneously. Hence, fast, and accurate fault detection and localization are essential to ensure system safety, reliability, and continuity of charging operations.

Traditionally, model-based, and signal-based diagnostic approaches have been used for fault detection in power converters^11^. Model-based methods rely on analytical redundancy, observers, or parity equations derived from converter models^12,13^. Although theoretically accurate, these approaches require precise system parameters and are sensitive to component tolerances and parameter drift. Signal-based methods, such as voltage and current analysis^14,15^, offer simpler implementation but often face difficulties in dealing with dynamic load variations and control-induced waveform distortions.

Recent advances in artificial intelligence (AI) have accelerated the use of data-driven diagnostic approaches. Machine learning and deep learning models, such as support vector machines, random forests, and convolutional neural networks (CNNs), have demonstrated strong capability in extracting transient features and achieving high fault diagnosis accuracy in power electronic systems^9,16–19^. More recently Graph Neural Networks (GNNs) have emerged as a powerful paradigm for modeling structural interactions among system components and distributed sensors. By representing systems as graphs, GNNs can effectively capture complex dependencies that are difficult to model using conventional approaches^20^.

In this context, physics-informed GNN frameworks have been introduced, such as the Physics-Informed Graph Modeling with Individualized Dynamics (PIGMind) method. This approach integrates physical priors into graph-based learning by embedding physics-derived indicators into the graph construction process, enabling the adjacency structure to reflect meaningful physical relationships. Such frameworks establish a promising direction for interpretable and physically consistent damage diagnosis in complex systems^21^.

Similarly, the Dynamic Causal Mechanism Learning Diagnostics Framework (DCMLDF) has been proposed to enhance diagnostic reliability by incorporating causal reasoning into data-driven models. Unlike conventional correlation-based approaches, DCMLDF aims to capture underlying fault mechanisms, thereby improving robustness under non-stationary operating conditions^22^.

Despite these advancements, GNN-based approaches still face several practical limitations. Constructing an appropriate graph representation from measurement data is often complex and may introduce additional modeling complexity. Moreover, their performance is highly dependent on the availability of large, balanced, and domain-specific datasets, which are typically limited in power electronic applications. In addition, there is a significant computational burden, particularly in large-scale or dynamic systems where graph structures evolve over time^20^.

In contrast, convolutional neural networks (CNNs) provide an efficient alternative for processing time–frequency representations of signals. By converting voltage measurements into scalograms, the fault diagnosis problem can be naturally formulated as an image-based classification task. CNNs can effectively extract localized spatiotemporal features without requiring explicit graph construction while offering lower computational complexity and simpler implementation. These advantages make CNN-based approaches more suitable for fault diagnosis in power converters^18,19^. However, their application to the DAB converters remains limited, while traditional methods are used for fault diagnosis in such isolated topologies^4,5,23,24^.

Most existing CNN-based studies on open-circuit fault diagnosis primarily formulate the problem as single-label (multi-class) classification, whether addressing single^18,25^ or simultaneous faults^19^, where each sample corresponds to only one fault class. This formulation inherently limits the ability to identify simultaneous open-circuit faults, which are common in multi-port systems such as DAB-based converters due to the strong coupling between converter arms and ports. Moreover, in such approaches, the number of classes increases combinatorially with the number of faulted switches, leading to increased model complexity and reduced scalability.

Furthermore, the literature has insufficiently explored several important aspects. These include the impact of sensor reduction on fault discriminability, the evaluation of diagnostic models under practical non-ideal operating conditions such as control signal delays (dead-time effects) and measurement noise, as well as the generalization capability to unseen multi-switch fault combinations. These limitations demonstrate the importance of a more flexible and robust diagnostic framework.

To address these challenges, this study proposes a multi-label fault localization approach for diagnosing open-circuit faults in a three-port DAB converter. The proposed framework is designed to effectively handle simultaneous faults, support reduced sensor configurations, and maintain high diagnostic performance under non-ideal operating conditions.

The main contributions of this work can be summarized as follows:

- The fault localization problem is formulated as a multi-label classification task. The proposed model enables detection and localization of multiple open-circuit faults and improves generalization to unseen fault combinations.
- A dataset construction strategy is developed, including all possible single and double open-circuit fault scenarios (79 cases), with variations in fault occurrence instants and battery state-of-charge levels.
- A multi-dimensional data fusion approach is introduced, where the time–frequency representations (scalograms) of six voltage sensors are concatenated into a three-dimensional input structure. Each sensor is treated as an independent channel, allowing the model to learn spatiotemporal correlations between multiple measurement points.
- The robustness of the proposed framework is validated under practical non-ideal conditions, including control signal delay (dead-time effects), sensor reduction, and measurement noise.

Finally, a deep learning-based diagnostic framework is implemented using a modified ResNet-18 architecture, where the input layer is adapted to multi-channel scalograms and the output layer employs a sigmoid activation function to support multi-label classification.

This paper is organized as follows: “System description” describes the three-port DAB converter topology and the measured signals used for fault diagnosis. “Dataset preparation” details the dataset generation procedure, including SOC variation, fault timing selection, and labeling strategy. “Feature extraction and preprocessing” presents the feature extraction and preprocessing process based on CWT. “Network architecture and training strategy” introduces the proposed multi-label classification model, including the modified ResNet-18 architecture and training strategy. “Evaluation metrics” defines the evaluation metrics used to assess diagnostic performance. “Results and discussion” provides comprehensive simulation results, including training behavior, overall fault localization accuracy, generalization to unseen fault combinations, comparison with multi-class classification, robustness to control signal delay, measurement noise and the effect of sensor reduction. “Real-time performance evaluation” provides a quantitative analysis of the computational burden and latency of the proposed framework. Finally, “Conclusion” concludes the paper and outlines directions for future research.

### System description

**Fig. 1:**
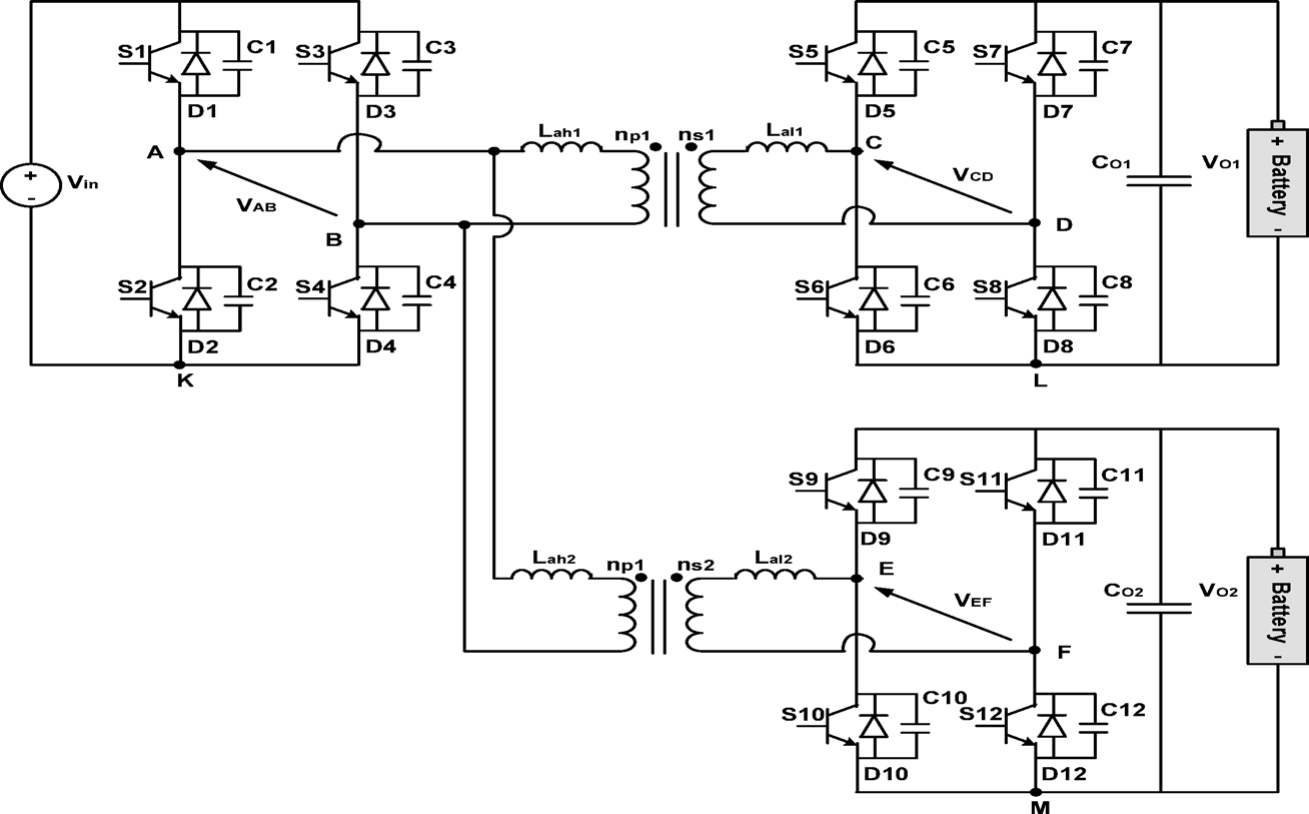
Three-port DAB converter used for simultaneous battery charging.

The system under study is a three-port isolated DC–DC converter derived from the DAB topology, as illustrated in Fig. 1. The converter enables the simultaneous charging of two batteries from a common DC input. On the primary side, a single full-bridge inverter $\:{(S}_{1}-{\mathrm{S}}_{4})$ generates a high-frequency square-wave voltage that excites two transformer primary windings. Each winding exhibits an associated leakage inductance $\:{(\mathrm{L}}_{\mathrm{a}\mathrm{h}1},{\mathrm{L}}_{\mathrm{a}\mathrm{h}2})$, which not only limits current transients but also facilitates soft-switching operation. On the secondary side, two independent full-bridge rectifier stages $\:{(S}_{5}-{\mathrm{S}}_{8}$ and $\:{S}_{9}-{\mathrm{S}}_{12})$ are connected to separate transformer secondary windings $\:{(\mathrm{n}}_{\mathrm{s}1},{\mathrm{n}}_{\mathrm{S}2})$. Their outputs are filtered by capacitors $\:{(\mathrm{C}}_{\mathrm{O}1},{\mathrm{C}}_{\mathrm{O}2})$ to provide isolated DC charging paths for two batteries $\:{(\mathrm{V}}_{\mathrm{O}1},{\mathrm{V}}_{\mathrm{O}2})$. Regulation of the power flow to each port is achieved by controlling the relative phase shift between the switching waveforms of the primary bridge and each corresponding secondary bridge^1^.

For monitoring and fault diagnosis purposes, the converter is instrumented at the midpoint of each bridge arm, producing six midpoint voltage measurements $\:{\mathrm{V}}_{\mathrm{A}}-{\mathrm{V}}_{\mathrm{F}}$. Each midpoint directly reflects the switching behavior of the two switches connected to its corresponding half-bridge leg; for example, $\:{\mathrm{V}}_{\mathrm{A}}$ corresponds to $\:{(S}_{1},{\mathrm{S}}_{2})$, $\:{\mathrm{V}}_{\mathrm{B}}$ corresponds to $\:{(S}_{3},{\mathrm{S}}_{4})$, and similarly for the remaining midpoints. Unlike line-to-line AC voltages, whose fault signatures may become indistinguishable under different open-circuit conditions, midpoint voltages preserve localized transient distortions, making them highly sensitive indicators for identifying individual switch faults^23,25^.

Due to the magnetic coupling among the transformer windings, fault-induced disturbances do not remain confined to their originating H-bridge; instead, they propagate through the coupled structure and influence the electrical behavior of the other bridges. The primary H-bridge has the strongest influence since it drives both secondary ports simultaneously. Consequently, any open-circuit fault in switches $\:{(S}_{1}-{\mathrm{S}}_{4})$ introduces measurable distortions not only in $\:{\mathrm{V}}_{\mathrm{A}}$ and $\:{\mathrm{V}}_{\mathrm{B}}$ but also across secondary-side midpoint voltages, enabling reliable detection even under reduced sensing configurations.

The first secondary bridge $\:{(S}_{5}-{\mathrm{S}}_{8})$ plays an important role in the converter’s control loop, thereby further amplifies the effect of its faults across the system and results in noticeable distortions in multiple midpoint voltages, not limited to $\:{\mathrm{V}}_{\mathrm{C}}$ and $\:{\mathrm{V}}_{\mathrm{D}}$. In contrast, the second secondary bridge $\:{(S}_{9}-{\mathrm{S}}_{12})$ operates as a slave port with weaker influence on the control feedback, which means that faults in its switches appear in $\:{\mathrm{V}}_{\mathrm{E}}$ and $\:{\mathrm{V}}_{\mathrm{F}}$. Consequently, removing sensors from this port causes a more significant drop in diagnostic accuracy compared with other ports.

This structural interaction between bridges, combined with the direct switch-to-sensor mapping, ensures that each open-circuit fault produces a unique voltage pattern distributed across the set of midpoint measurements. These patterns form the basis of the proposed feature extraction and multi-label fault localization methodology.

### Dataset preparation

The dataset used to train and evaluate the proposed fault localization model was generated through time-domain simulations of the three-port DAB converter under various operating and fault conditions. The data generation process systematically varied two system parameters, the SOC of the batteries and the time intervals (T₁, T₂) representing the fault occurrence instants, to capture a wide range of converter behavior.

The SOC values were selected between 15% and 90%, distributed over 25 equally spaced levels. The total simulation time of each run was 2.5 ms, which was divided into five equal intervals. Within each interval, the values of T₁ and T₂ for simultaneous faults were chosen randomly to ensure that faults occurred at various positions along the switching period. This stochastic selection approach guarantees that the resulting dataset contains sufficient temporal diversity and avoids bias toward specific timing conditions.

To calculate the total number of generated samples for training, different combinations of SOC and timing values were used depending on the fault type:

- Individual fault:

For each SOC level, five random T₁ values were generated within the defined time intervals. Considering 20 SOC samples in the training set, the procedure results in 100 training samples per single-fault case.

- Two simultaneous faults:

For each SOC level, two independent time points, T₁ and T₂, were randomly selected from the five intervals. Hence, the total number of training samples reaches 500 per two- switch fault case.

- Healthy condition:

Only SOC variations were considered, producing 20 training samples.

The same generation procedure was applied for the test dataset using five unseen SOC levels, yielding 125 samples for two-switch faults, 25 for single-switch faults, and five for the healthy case. The random variation of fault occurrence instants, along with the use of unseen SOC levels for testing, ensures data diversity and reduces the risk of overfitting, thereby improving the generalization capability of the model.

All simulations were performed over a 2.5 ms window, and the midpoint voltages $\:{\mathrm{V}}_{\mathrm{A}}-{\mathrm{V}}_{\mathrm{F}}$ were recorded at a sampling frequency of 10 MHz, resulting in approximately 25,000 samples per signal channel. The final datasets were stored in separate training and testing folders to ensure full data independence during model evaluation.

To construct the labeled datasets, each sample was encoded as a 12-bit multi-label vector, where “1” indicates the presence of a fault in the corresponding switch and “0” represents a healthy condition. In total, 79 distinct fault scenarios were studied, including 12 single-switch faults, 66 simultaneous two-switch faults, and one healthy condition. Table 1 summarizes all studied open-circuit fault categories, including single- and two-switch combinations, their physical locations, and representative 12-bit label encodings used for multi-label classification. Table 2 presents a summary of the generated training and testing datasets, including all fault types and the healthy condition.

**Table 1:** Summary of all studied fault categories in the three-port DAB converter.

Category	Fault location	Switches involved	Technical description	No. of classes	Representative 12-bit Label (S1–S12)
Healthy condition	–	–	Converter operates normally with no open-circuit fault.	1	[0 0 0 0 0 0 0 0 0 0 0 0]
Individual open-circuit faults	Primary side	(S1-S4)	Single fault on the main inverter bridge driving the transformer primaries.	4	S2 → [0 1 0 0 0 0 0 0 0 0 0 0]
	Secondary side one (Port 1)	(S5-S8)	Single fault on the first secondary bridge supplying Battery (1).	4	S6 → [0 0 0 0 0 1 0 0 0 0 0 0]
	Secondary side two (Port 2)	(S9-S12)	Single fault on the second secondary bridge supplying Battery (2).	4	S10 → [0 0 0 0 0 0 0 0 0 1 0 0]
Simultaneous open-circuit faults	Same bridge, same phase	(S1, S2), (S3, S4), (S5, S6), (S7, S8), (S9, S10), (S11, S12)	Two adjacent switches on the same leg fail simultaneously, disabling one bridge arm.	6	S1 + S2 → [1 1 0 0 0 0 0 0 0 0 0 0]
	Same bridge, diagonal switches	(S1, S4), (S2, S3), (S5, S8), (S6, S7), (S9, S12), (S10, S11)	Diagonal pair within the same H-bridge fails simultaneously, creating cross-leg open circuits.	6	S1 + S4 → [1 0 0 1 0 0 0 0 0 0 0 0]
	Same bridge, upper switches	(S1, S3), (S5, S7), (S9, S11)	Both upper switches in one bridge fail.	3	S1 + S3 → [1 0 1 0 0 0 0 0 0 0 0 0]
	Same bridge, lower switches	(S2, S4), (S6, S8), (S10, S12)	Both lower switches in one bridge fail.	3	S2 + S4 → [0 1 0 1 0 0 0 0 0 0 0 0]
	Cross-bridge (Primary ↔ Secondary 1)	(S1-S4) x (S5-S8)	Faults involving one primary and one secondary (1) switch occurring simultaneously.	16	S1 + S5 → [1 0 0 0 1 0 0 0 0 0 0 0]
	Cross-bridge (Primary ↔ Secondary 2)	(S1-S4) x (S9-S12)	Faults involving one primary and one secondary (2) switch occurring simultaneously.	16	S2 + S10 → [0 1 0 0 0 0 0 0 0 1 0 0]
	Cross-bridge (Secondary 1 ↔ Secondary 2)	(S5-S8) x (S9-S12)	Faults involving one switch from each secondary bridge.	16	S5 + S9 → [0 0 0 0 1 0 0 0 1 0 0 0]

**Table 2:** Summary of training and testing datasets for all fault categories.

Dataset description	Training dataset	Testing dataset
Healthy status	20	5
Individual fault	1200	300
Two simultaneous faults	33,000	8250
Total dataset	34,220	8555

It is important to note that the dataset is intentionally unbalanced, with a larger number of samples allocated to simultaneous fault scenarios due to their higher complexity and variability compared to single-fault and healthy conditions.

### Feature extraction and preprocessing

To enable the use of deep convolutional neural networks for multi-label fault localization, the time-domain midpoint voltage signals $\:{\mathrm{V}}_{\mathrm{A}}-{\mathrm{V}}_{\mathrm{F}}$ were transformed into two-dimensional time–frequency representations using the CWT. CWT provides localized information in both time and frequency domains, allowing transient signatures caused by open-circuit faults to be clearly distinguished from normal operating conditions.

The CWT of a signal $\:x\left(t\right)$ is defined as:

1 $$\:{CWT}_{x}\left(s,\tau\:\right)=\frac{1}{\sqrt{\left|s\right|}}{\int\:}_{-\infty\:}^{\infty\:}x\left(t\right){\:\psi\:}^{*}\left(\frac{t-\tau\:}{s}\right)dt\:$$

Where $\:s$ and $\:\tau\:$ denote the scale and time shift parameters respectively, and *$\:\psi\:\left(t\right)$ is the mother wavelet^18,26,27^.

In this work, the complex Morlet wavelet was selected due to its superior time–frequency localization properties and its effectiveness in capturing short-duration distortions typical of open-circuit switch faults in power converters^25^.

**Fig. 2:**
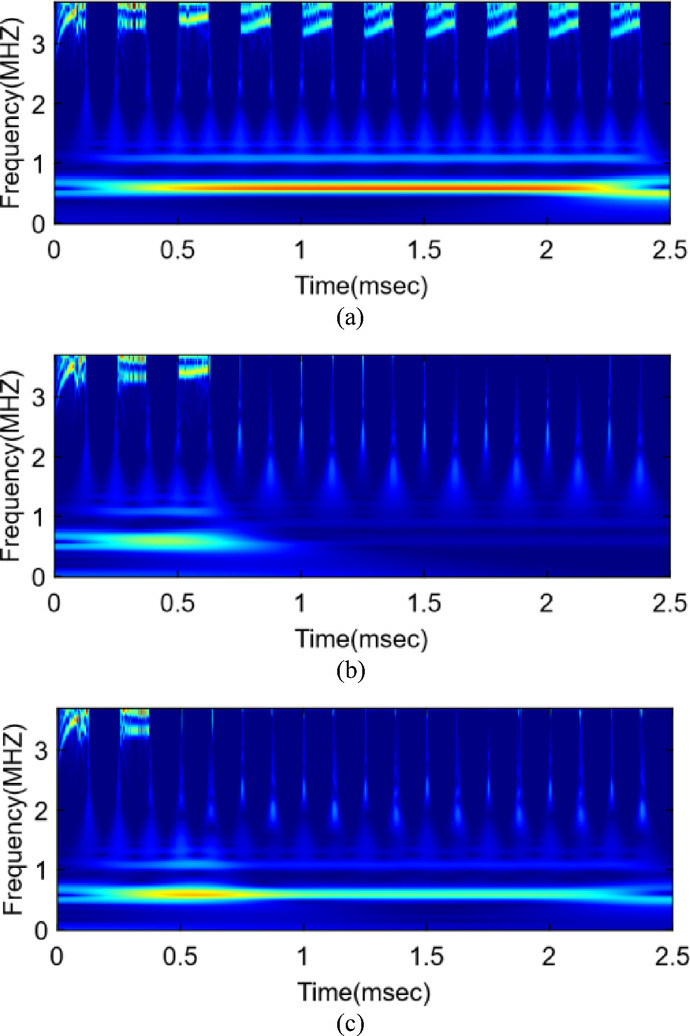
CWT scalograms represent midpoint voltage $\:{V}_{A}$​ under (**a**) healthy, (**b**) single-switch fault(S1), and (**c**) double-switch fault conditions (S1 + S4).

The CWT coefficients were computed over the entire 2.5 ms simulation window, with frequency scales chosen to represent the dominant switching and harmonic components of the converter. The resulting scalograms were converted into magnitude images corresponding to the absolute values of the wavelet coefficients. Figure 2 illustrates representative examples of CWT scalograms generated for both healthy and faulty classes.

To obtain compact and uniform feature maps, the wavelet coefficient matrices were downsampled by averaging every 50 samples along the time axis, while maintaining the original number of frequency scales to preserve spectral resolution and fault-related features. This resulted in images with a fixed dimension of (118 × 500) for each signal channel. Subsequently, these wavelet coefficient matrices from the six sensor signals were concatenated into a three-dimensional array to form multi-sensor composite images. For each sample, the six CWT channels were stacked along the third dimension, producing a six-channel representation of size (118 × 500 × 6) that captures correlated time–frequency patterns across all measurement points. These combined feature arrays were stored in separate subfolders corresponding to different fault categories.

To ensure consistent feature scaling and improve network convergence, channel-wise normalization was applied across all training samples. The mean and standard deviation of each channel were computed from the entire training set and stored as normalization parameters. Each CWT image was then standardized according to:

2 $$\:{X}^{{\prime\:}}=\frac{X-{\mu\:}_{ch}}{{\sigma\:}_{ch}}$$

where $\:{\mu\:}_{ch}$ and $\:{\sigma\:}_{ch}$ denote the mean and standard deviation of channel $\:ch$, respectively.

The same normalization parameters were then applied to the testing dataset to maintain statistical consistency and prevent information leakage.

This preprocessing effectively transforms the raw time-domain voltage signals into normalized multi-channel CWT scalograms, preserving the transient and spectral features essential for accurate open-circuit fault localization using convolutional neural networks.

### Network architecture and training strategy

A deep convolutional neural network based on the ResNet-18 architecture was employed to perform multi-label classification of open-circuit faults in the three-port DAB converter. The network was initialized without pretrained weights to allow domain-specific feature learning from the CWT scalogram dataset. The input layer was replaced by a custom image input layer of size.

(118 × 500 × 6), corresponding to the six-channel CWT feature maps obtained from the midpoint voltage signals $\:{\mathrm{V}}_{\mathrm{A}}-{\mathrm{V}}_{\mathrm{F}}$.

**Fig. 3:**
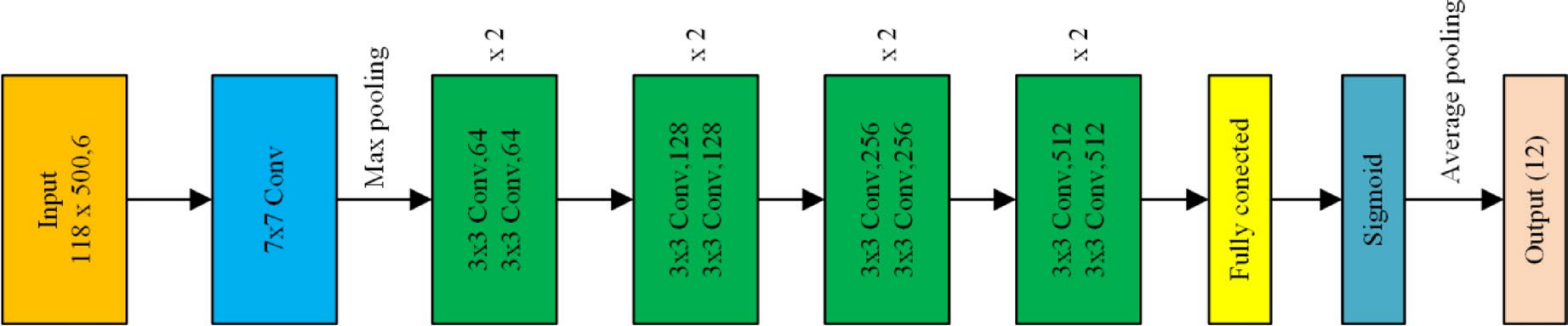
Modified ResNet-18 for multi-label fault localization.

The original ResNet-18 classification layers (fc1000, softmax, and classification output) were removed and replaced with a new sequence consisting of a fully connected layer with 12 neurons, followed by a sigmoid activation layer to adapt the model for multi-label classification. Each neuron corresponds to one of the twelve converter switches $\:{(\mathrm{S}}_{1}-{\mathrm{S}}_{12})$, generating independent activation probabilities that indicate the prospect of open-circuit faults. Figure 3 illustrates the modified ResNet-18 architecture used for multi-label fault localization, showing the customized input layer, the residual blocks, and the final sigmoid-based multi-label output layer^28^.

The network was trained using a binary cross-entropy loss function, defined as:

3 $$\:\mathcal{L}=-\frac{1}{N}\sum\:_{i=1}^{N}\left[{y}_{i}\mathrm{log}\left({\widehat{y}}_{i}\right)+\left(1-{y}_{i}\right)\mathrm{log}\left(1-{\widehat{y}}_{i}\right)\right]$$

where $\:{y}_{i}$and $\:{\widehat{y}}_{i}$ denote the true and predicted fault labels, respectively.

The model parameters were optimized using the Adam optimizer with a fixed learning rate of 0.0001 and a mini-batch size of 128. All simulation models were run on a workstation with an Intel (R) Core (TM) i7-9850 H CPU @ 2.60 GHz, 32.0 GB installed RAM, and an NVIDIA Quadro RTX 3000 Graphics Card.

To improve generalization, the dataset was randomly split into 80% training and 20% validation. During each epoch, the model parameters were updated based on the training loss and subsequently evaluated on the validation set using the F1-score as the main performance metric. An early-stopping criterion was implemented to prevent overfitting and ensure generalization. The training process was automatically terminated if the validation F1-score failed to improve by at least 0.1% over several consecutive epochs. The best-performing model weights, corresponding to the highest F1-score, were saved for final evaluation.

After training, the best model was evaluated on a separate test dataset that included all fault scenarios. Performance assessment was conducted using the macro- and micro-averaged metrics detailed above, enabling a balanced evaluation across frequent and rare fault types.

**Fig. 4:**
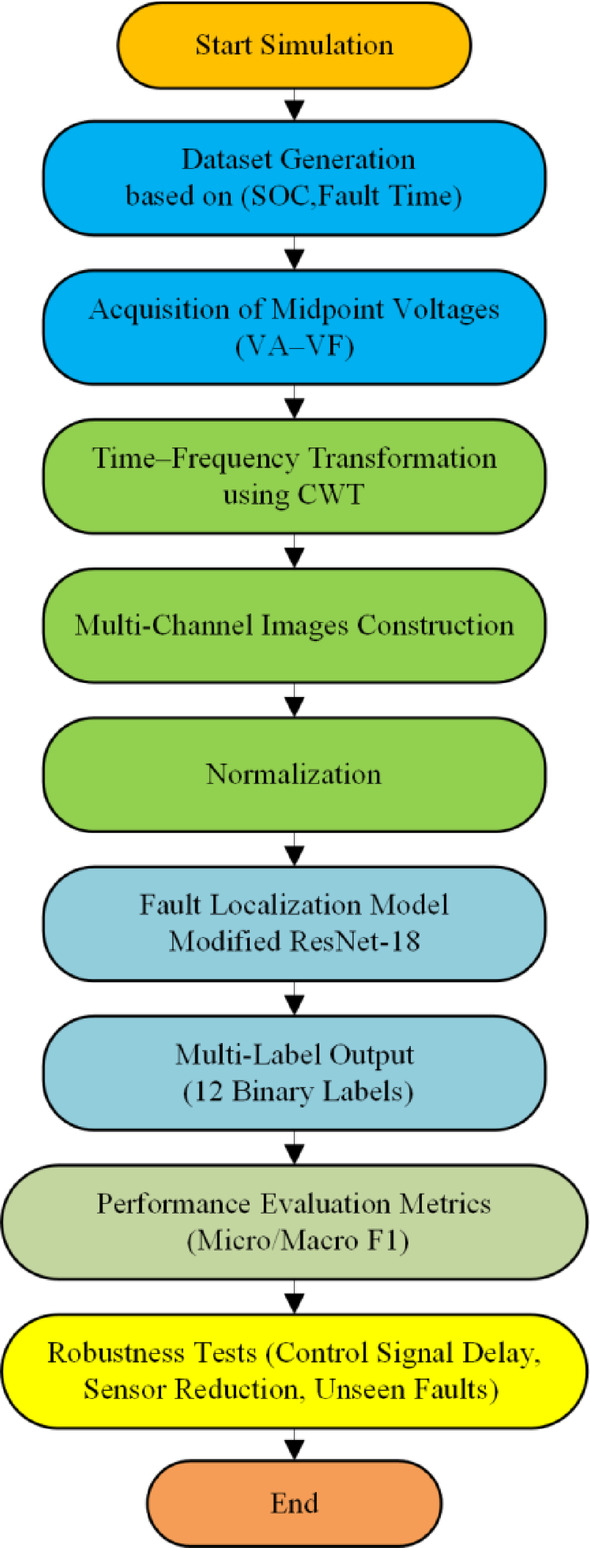
Overall flow chart of the proposed framework in this paper.

This training strategy enables the modified ResNet-18 architecture to effectively learn multi-channel spectral representations from CWT scalograms, resulting in reliable and accurate localization of both individual and simultaneous open-circuit faults in the multi-port DAB converter. Figure 4 shows the flowchart of the proposed multi-label fault localization model.

### Evaluation metrics

The performance of the proposed multi-label fault localization network can be assessed with both macro-averaged and micro-averaged metrics. These metrics are commonly utilized in multi-label classification and imbalanced data contexts, as they provide a balanced and comprehensive assessment of classification performance^29,30^. In particular, the macro-averaged metrics evaluate each class independently, ensuring that classes with fewer samples are not dominated by those with larger sample sizes.

For each label $\:i$, the precision $\:{P}_{i}$ and recall $\:{R}_{i}$ were calculated as:

4 $$\:{P}_{i}=\frac{T{P}_{i}}{T{P}_{i}+F{P}_{i}}\:,\:$$

5 $$\:{R}_{i}=\frac{T{P}_{i}}{T{P}_{i}+F{N}_{i}}\:$$

where $\:T{P}_{i}$, $\:F{P}_{i}$, and $\:F{N}_{i}$ denote the true positive, false positive, and false negative counts, respectively, for label $\:i$.

The per-label F₁-score $\:{F}_{1i}$ was then computed as:

6 $$\:{F}_{1i}=\frac{2{P}_{i}{R}_{i}}{{P}_{i}+{R}_{i}}\:$$

The macro-averaged F₁-score was obtained as the arithmetic mean across all $\:L$ labels:

7 $$\:{F}_{1}^{Macro}=\frac{1}{L}\sum\:_{i=1}^{L}{F}_{1i}\:$$

while the micro-averaged F₁-score was calculated globally by aggregating all $\:TP$, $\:FP$, and $\:FN$ across labels before applying the same formula:

8 $$\:{F}_{1}^{Micro}=\frac{2\sum\:TP}{2\sum\:TP+\sum\:FP+\sum\:FN}\:$$

These metrics jointly provide a balanced evaluation of both overall and per-label performance, ensuring that the network’s ability to detect rare and frequent fault types is accurately assessed.

## Results and discussion

This section presents the simulation results obtained using the proposed multi-label fault localization framework. The simultaneous charging system is modeled and simulated in MATLAB/Simulink, while the data preprocessing and classification are implemented in MATLAB coding. To ensure reproducibility and provide a clear description of the operating conditions, the main electrical parameters of the simulated three-port DAB converter are summarized in Table 3^31^.

**Table 3:** Electrical parameters of the simulated model.

Parameter	Value
High-side voltage $\:{(\mathrm{V}}_{\mathrm{i}\mathrm{n}})$	$\:360\:\mathrm{V}$
Low-side voltages $\:{(\mathrm{V}}_{\mathrm{o}1},{\mathrm{V}}_{\mathrm{o}2})$	$\:60\:\mathrm{V}$
Nominal power	$\:6.5\:\mathrm{k}\mathrm{W}$
Switching frequency $\:{(f}_{sw}$)	$\:4\:\mathrm{k}\mathrm{H}\mathrm{z}$
Turns ratio ($\:{n}_{p1}/{n}_{s1}-\:{n}_{p1}/{n}_{s2})$	$\:6/1$
High-side auxiliary inductances $\:{(\mathrm{L}}_{\mathrm{a}\mathrm{h}1},{\mathrm{L}}_{\mathrm{a}\mathrm{h}2})$	$\:5\:{\upmu\:}\mathrm{H}$
Low-side auxiliary inductances $\:{(\mathrm{L}}_{\mathrm{a}\mathrm{l}1},{\mathrm{L}}_{\mathrm{a}\mathrm{l}2})$	$\:0.14\:{\upmu\:}\mathrm{H}$
Output capacitances $\:{(\mathrm{C}}_{\mathrm{o}1},{\mathrm{C}}_{\mathrm{o}2})$	$\:22\:\mathrm{m}\mathrm{F}\:$
Primary resistances	$\:34\mathrm{m}{\Omega\:}$
Primary leakage inductances	$\:8.1\:{\upmu\:}\mathrm{H}$
Secondary resistances	$\:0.94\:\mathrm{m}{\Omega\:}$
Secondary leakage inductances	$\:0.225\:{\upmu\:}\mathrm{H}$

### Training and validation performance

The proposed multi-label fault localization network was evaluated across twenty epochs using the prepared training and validation datasets.

**Fig. 5:**
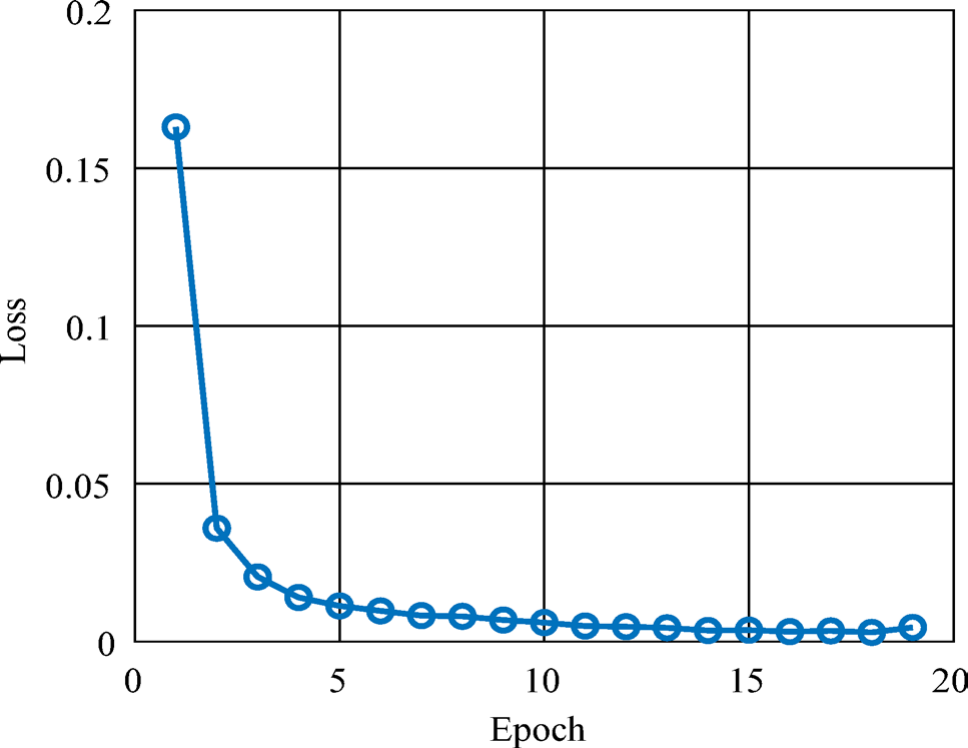
Training loss of ResNet-18-based multi-label fault localization network.

Figure 5 illustrates the evolution of the training loss, which rapidly decreased from approximately 0.16 to below 0.01 within the first five epochs, indicating rapid convergence and efficient gradient optimization. The loss then stabilized around 0.003, demonstrating that the model effectively minimized the error without exhibiting instability or overfitting.

**Fig. 6:**
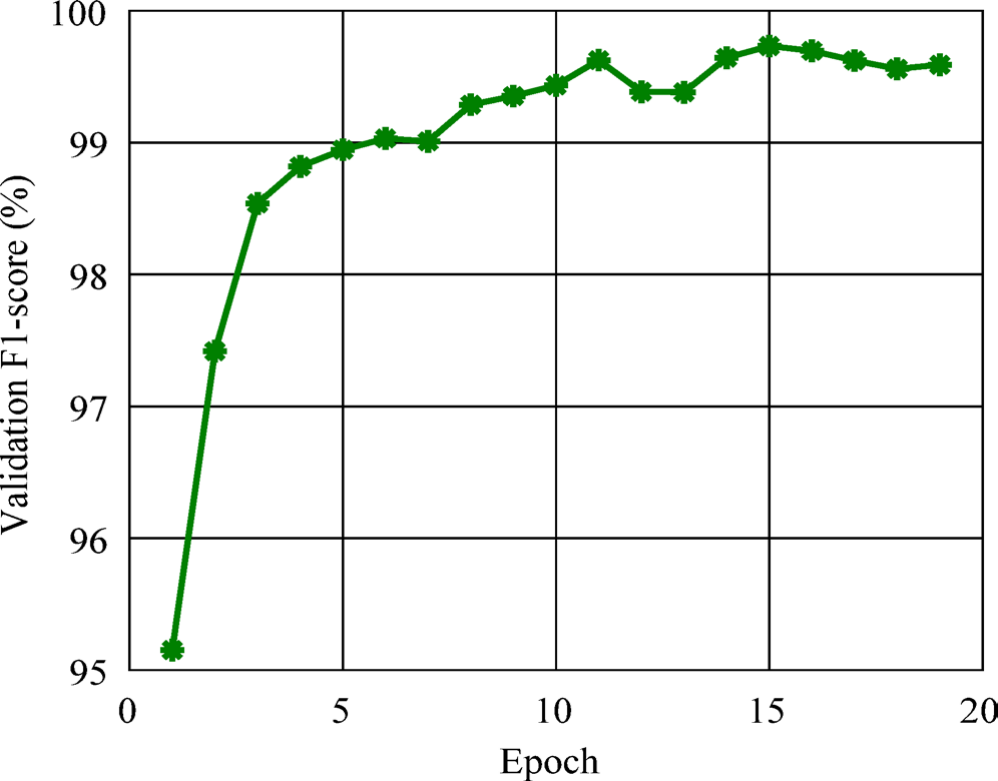
Training validation F₁-score.

Figure 6 shows the validation F₁-score progression, which increased steadily from about 95% in the early epochs to above 99% after epoch ten. At epoch fifteen, the network had the best validation performance, with an F₁-score of 99.73% and a training loss of 0.003. At this point, the early stopping criterion was automatically triggered, indicating that the model had reached optimal generalization without further improvement on the validation set.

The combination of a rapidly decreasing loss and high validation performance demonstrates the model’s strength and its ability to extract discriminative fault-related features from the CWT-based representations. These results confirm that the proposed ResNet-18 model efficiently generalizes across varying operating conditions and fault types.

### Fault localization performance

After the training process, the proposed multi-label ResNet-18 model was evaluated on an independent test dataset containing all scenarios, including healthy cases, single-switch faults, and simultaneous two-switch faults.

The model achieved a micro-averaged precision of 0.9975, a recall of 0.9910, and an F₁-score of 0.9942, indicating that the network effectively distinguishes fault patterns across all labels while maintaining a low rate of false positives and missed detections. The macro-averaged results show identical values, confirming that the classifier performs uniformly across both frequent and less common fault types, without bias toward specific fault locations.

Table 4 summarizes the per-label precision, recall, and F₁-scores for all twelve switch fault labels. The high precision and recall values across all labels indicate that the model successfully learns time–frequency features from the CWT scalograms, achieving near-perfect multi-label fault localization accuracy for both individual and simultaneous open-circuit faults in the three-port DAB converter.

**Table 4:** Per-label precision, recall, and F₁-score for twelve fault labels.

Label	Precision	Recall	F1-score
01	1.0000	0.981	0.991
02	1.0000	1.000	1.000
03	0.999	0.991	0.995
04	1.000	1.000	1.000
05	1.000	0.999	1.000
06	1.000	0.999	1.000
07	1.000	0.993	0.996
08	1.000	0.999	1.000
09	0.979	0.982	0.981
10	0.999	0.986	0.992
11	0.998	0.998	0.998
12	0.994	0.962	0.978

To evaluate the performance of the proposed model, several well-established deep learning architectures, including AlexNet, VGG16, MobileNetV2, and EfficientNet-B0, were employed for comparison. These models rely on converting signals into time–frequency representations, which have been widely adopted in fault diagnosis tasks.

The selected models are based on CNNs, which are highly effective in extracting discriminative features from image-based representations. However, they differ in terms of complexity, computational efficiency, and architectural design. AlexNet and VGG16 are considered classical deep learning models that emphasize deep feature extraction and have demonstrated strong performance in various fault diagnosis applications^32^. In contrast, MobileNetV2 is a lightweight architecture designed to reduce computational cost while maintaining competitive accuracy, making it suitable for real-time and embedded systems^33^. EfficientNet-B0 represents a more recent advancement, introducing compound scaling to balance network depth, width, and resolution, thereby achieving improved accuracy and efficiency trade-offs^34^.

Furthermore, these architectures were originally designed for single-label classification tasks. Therefore, their final classification layers were modified to support multi-label classification, enabling the detection of multiple simultaneous faults. Table 5 presents the comparative performance of the proposed model and the different architectures.

**Table 5:** Performance of a multi-label fault localization model achieved using different deep learning architectures.

Model	Precision	Recall	F1-score	Average inference time per sample(ms)
ResNet18(Proposed)	0.997	0.991	0.994	9.66
AlexNet	0.980	0.977	0.978	6.6
MobileNetV2	0.954	0.908	0.929	29.1
EfficientNet	0.964	0.891	0.923	43.4
VGG16	0.926	0.941	0.933	11

In addition to classification performance, the computational efficiency of the models was evaluated based on the average inference latency per sample (batch size = 1) to simulate an online diagnostic environment. As shown in Table 5, AlexNet achieved the fastest inference time, 6.6 ms per sample, followed by the proposed ResNet18-based model, 9.66 ms per sample. Although MobileNetV2 and EfficientNet-B0 are designed as efficient architectures, their inference times were higher, at 29.1 ms and 43.4 ms per sample, respectively. VGG16 required 11 ms per sample.

Despite not being the fastest model, the proposed ResNet18 achieved the best overall performance by providing the highest classification accuracy while maintaining a low inference time. This demonstrates that the proposed model offers an optimal balance between accuracy and computational efficiency, making it highly suitable for fault diagnosis applications.

### Generalization of unseen simultaneous faults

In this section, to evaluate the generalization capability of the proposed multi-label diagnostic model, an additional test was conducted using fault scenarios that were not included during training. While the model was trained exclusively on single-switch and two-switch open-circuit faults, the extended test set additionally incorporated three-switch simultaneous faults, which represent different failure patterns and are more complex.

The extended test dataset contained 299 scenarios comprising 12 single-switch faults, 66 two-switch simultaneous faults, 220 three-switch simultaneous faults, and one healthy condition for a total of 2275 test samples.

Despite never using three-switch combinations during training, the model achieved high generalization performance, obtaining a micro-F₁-score of 0.8464 and a macro-F₁-score of 0.8441. These results demonstrate that the proposed network successfully learned the time–frequency representations that extend to more complex fault patterns.

Label precision, recall, and F₁-scores are summarized in Table 6. Overall, the results confirm that the proposed model exhibits robust generalization to unseen multi-switch fault combinations, demonstrating its practicality for scenarios where unexpected or rare simultaneous faults may occur.

**Table 6:** Per-label precision, recall, and F₁-score for unseen three-switch simultaneous faults.

Label	Precision	Recall	F1-score
01	0.973	0.804	0.881
02	0.984	0.820	0.894
03	0.955	0.800	0.871
04	0.985	0.857	0.917
05	0.992	0.804	0.888
06	0.992	0.776	0.871
07	0.986	0.763	0.860
08	0.963	0.800	0.874
09	0.917	0.635	0.751
10	0.972	0.613	0.752
11	0.924	0.613	0.737
12	0.916	0.673	0.776

### Comparison with multi-class classification

In this section, to verify the effectiveness of the proposed multi-label classification model, an equivalent multi-class ResNet-18 classifier was implemented using the same CWT scalogram dataset under identical training parameters.

In the multi-class classification model, each sample is assigned to one of 79 classes representing the healthy state, 12 single-switch faults, or 66 two-switch simultaneous faults. The model uses softmax activation with categorical cross-entropy loss, restricting the network to output exactly one class per input sample. In contrast, the proposed multi-label model uses sigmoid activation and binary cross-entropy loss, enabling simultaneous prediction of multiple fault labels. This structural difference allows the multi-label network to naturally represent simultaneous faults, making it inherently better suited for DAB-based systems where open-circuit faults frequently occur in combinations.

Table 7 presents the performance comparison between the two models. The multi-class classifier achieved a macro-F₁ score of 0.963 and a micro-F₁ score of 0.987, both of which are lower than the proposed multi-label model (0.9942 for both metrics). This corresponds to a 3.1% drop in macro-F₁ and a 0.7% drop in micro-F₁, translating to a sixfold increase in overall misclassification error and a doubling of global prediction error.

**Table 7:** Performance comparison between multi-class and multi-label models.

Model	Macro -F_1_	Micro-F_1_
Multi-Class ResNet-18	0.963	0.987
Proposed Multi-Label ResNet-18	0.9942	0.9942

This performance gap between the two models is expected to widen further if the number of samples is distributed equally across fault types, since the multi-class classification treats every unique fault combination as a distinct class, leading to sparse data representation.

These results confirm that the multi-label classification provides a more flexible and realistic representation of open-circuit fault behavior in the multi-port DAB converter, effectively capturing both individual and simultaneous faults that a traditional multi-class classifier cannot distinguish.

### Robustness analysis under control signal delay (dead-time effects)

To assess the robustness of the proposed multi-label fault localization model, an additional experiment was conducted in which a delay was inserted into the control gate signals during testing while the trained model remained unchanged, as described in Subsection A. These delays simulate practical timing uncertainties in power electronic converters, particularly dead-time effects, which may distort voltage waveforms and alter the transient signatures used for fault diagnosis.

The experimental design has been extended by incorporating realistic dead-time variations in the control signals. Specifically, the dead-time values are selected within the range of 1 µs to 5 µs, which is consistent with practical implementations^35^.

In this analysis, the dead time is simultaneously applied to all bridge legs, reflecting practical converter operation where timing delays are inherently present across all switching devices. For each dead-time value, an independent test dataset is generated to enable a clear and controlled evaluation of the model performance under different timing conditions.

The test samples are constructed for all operating conditions, including healthy, single-fault, and double-fault cases. For each class, five different SOC levels are distributed between 15% and 90%, and five distinct fault-occurrence instants are considered within the 2.5 ms simulation time, ensuring temporal diversity and avoiding bias toward specific switching instants. In addition, the number of samples is kept equal across all classes to ensure a balanced and unbiased evaluation.

Dead-time variations are applied to the healthy and single-fault cases, while double-fault scenarios are evaluated under nominal timing conditions. This design allows isolating the impact of timing distortions without introducing additional complexity due to overlapping fault signatures.

**Fig. 7:**
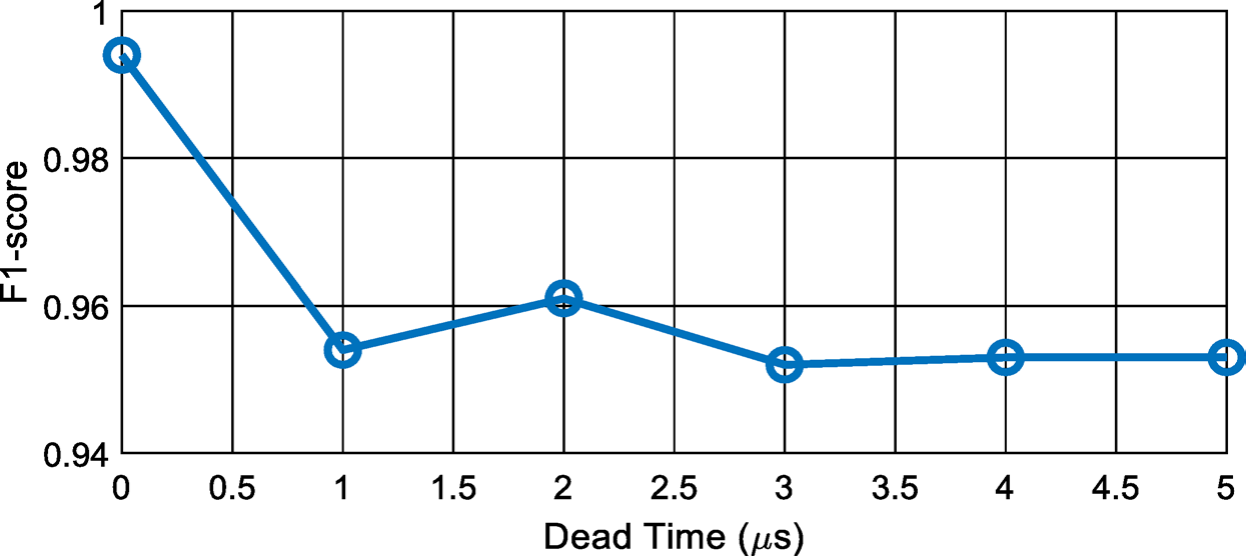
Mean F1-score vs. dead time.

Figure 7 illustrates the effect of dead-time variations on the diagnostic performance of the proposed model, including the baseline case without dead time. The highest F1-score of approximately 0.99 is achieved under ideal conditions (0 µs), while slightly lower values ranging from 0.952 to 0.961 are observed when dead time is introduced.

Despite this slight reduction, the performance degradation remains minimal across the entire range of dead-time values. The narrow variation in F1-score indicates that the proposed model is insensitive to timing distortions introduced by dead time. This behavior suggests that the extracted features preserve their discriminative capability even under waveform distortions, confirming the robustness and practical applicability of the proposed approach in real converter environments.

However, the performance degradation remains minimal across the entire range of dead-time values, with the F1-score varying within a narrow band. This indicates that the proposed model is insensitive to timing distortions introduced by dead time. The results confirm that the extracted features preserve their discriminative capability even under non-ideal switching conditions, demonstrating strong robustness for practical applications.

### Effect of sensor reduction on fault localization accuracy

To assess the dependence of the proposed fault localization model on both the number and placement of voltage sensors, a systematic sensor reduction study was conducted. The objective is to evaluate the trade-off between diagnostic accuracy and sensing complexity, which is critical for practical implementations.

**Fig. 8:**
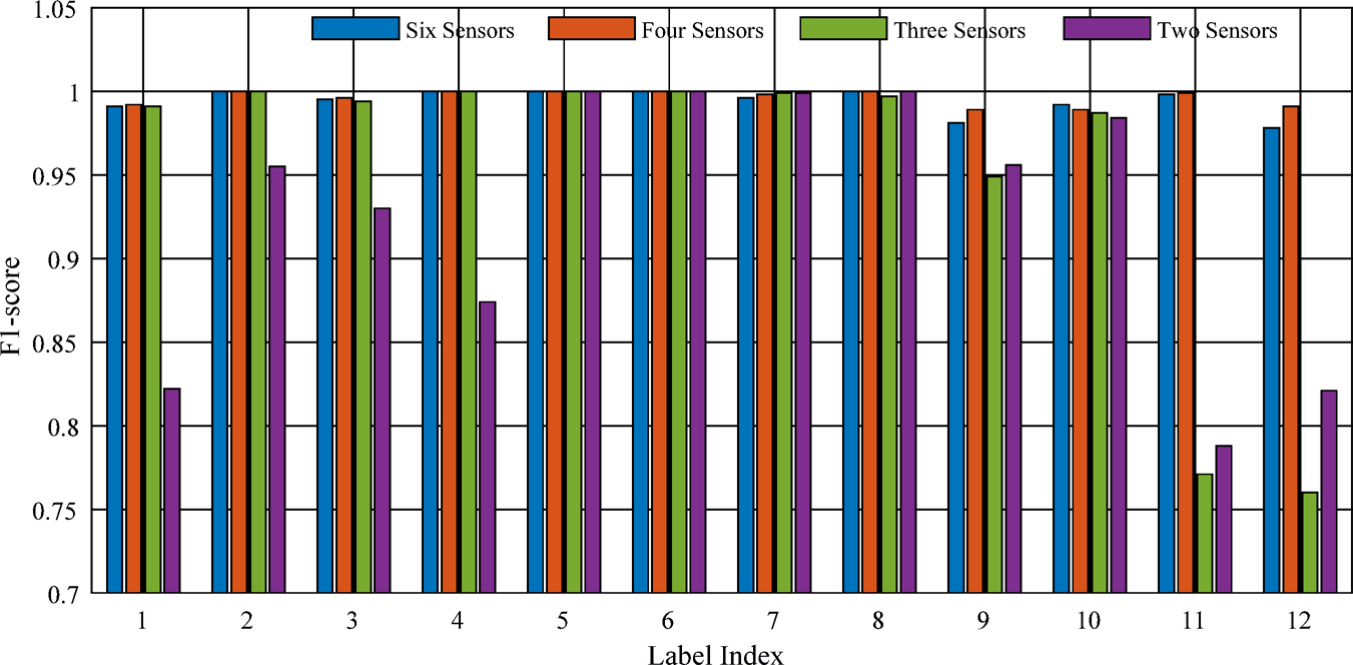
Comparison of per-label F₁-scores under different voltage sensor configurations.

The baseline configuration employs all six midpoint voltage measurements $\:{(\mathrm{V}}_{\mathrm{A}}-{\mathrm{V}}_{\mathrm{F}})$. Reduced sensor configurations are then constructed using four, three, and two sensors. The selection of these sensors is not arbitrary; rather, it is based on their physical locations within the three-port DAB converter, allowing a structured evaluation of how sensor placement influences fault discrimination capability. A comparison of the considered sensing configurations is illustrated in Fig. 8.

The results indicate that the proposed model maintains high diagnostic accuracy even with a reduced number of sensors. Removing sensors $\:{\mathrm{V}}_{\mathrm{B}}$ and $\:{\mathrm{V}}_{\mathrm{D}}$ results in only a marginal decrease in the F_1_-score, suggesting that the remaining sensors still capture the dominant fault signatures due to the strong electrical coupling between converter bridges.

In contrast, removing sensors associated with the third H-bridge leads to a more noticeable performance degradation. This is because the fault signatures of switches $\:{\mathrm{S}}_{11}$ and $\:{\mathrm{S}}_{12}$ are primarily reflected in the $\:{\mathrm{V}}_{\mathrm{F}}$ measurement, making this sensor more critical for accurate fault localization.

**Fig. 9:**
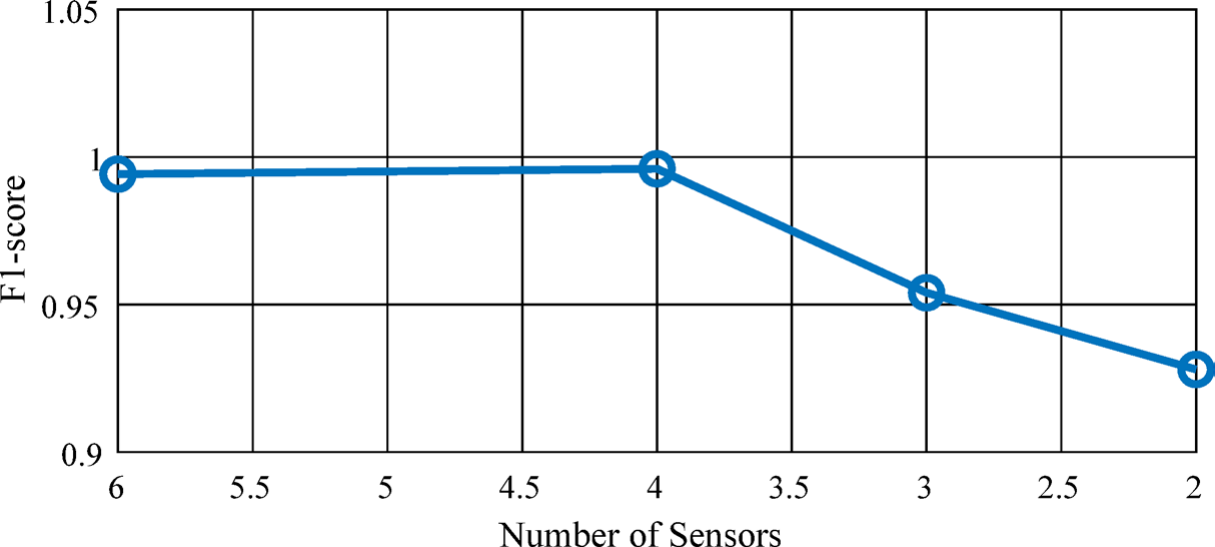
Mean F₁-score as the number of sensors decreases.

As shown in Fig. 9, the micro-F_1_ score decreases from 0.9942 with all six sensors to 0.954 when using three sensors $\:{(\mathrm{V}}_{\mathrm{A}},{\mathrm{V}}_{\mathrm{C}},{\mathrm{V}}_{\mathrm{E}})$, and further to 0.927 when only two sensors $\:({\mathrm{V}}_{\mathrm{C}},{\mathrm{V}}_{\mathrm{E}})$ are retained. Despite this reduction, the model continues to achieve reliable fault localization performance, demonstrating its robustness and suitability for sensor-constrained environments.

The analysis confirms that both the number and the placement of sensors play a crucial role in diagnostic performance, while also highlighting the ability of the proposed method to operate effectively with a limited sensing configuration.

### Robustness analysis under measurement noise

To evaluate the robustness of the proposed fault diagnosis framework under realistic measurement conditions, the effect of measurement noise is investigated. Electromagnetic interference (EMI) and switching noise often affect voltage measurements in practical power electronic systems, which may distort signal characteristics and degrade diagnostic performance.

To simulate realistic operating conditions, noisy datasets at different signal-to-noise ratio (SNR) levels (40 dB and 30 dB) were generated using MATLAB. These SNR values are consistent with typical noise levels observed in power electronic converters^36^. The generated datasets include all operating conditions, covering healthy, single-fault, and simultaneous fault scenarios, as listed in Tables 1 and 2.

Additive white Gaussian noise (AWGN) was independently added to each sensor signal to emulate uncorrelated measurement disturbances across different channels. The noisy signals were then processed using the same pipeline as the original data, including CWT and feature extraction. The trained deep learning model was subsequently applied without any retraining or parameter adjustment to ensure a fair robustness evaluation.

Table 8 presents the diagnostic performance of the proposed framework under different noise conditions. The clean signals yield the highest performance, achieving an F_1_-score of 0.994. When additive white Gaussian noise is introduced at an SNR of 40 dB, only a slight degradation in performance is observed, with the F_1_-score remaining as high as 0.979. This indicates strong robustness under low-noise conditions.

**Table 8:** Diagnostic performance under different SNR levels.

Condition	Precision	Recall	F_1_-Score
Clean	0.997	0.991	0.994
40 dB	0.983	0.974	0.979
30 dB	0.939	0.924	0.931

At a lower SNR of 30 dB, which represents a more challenging and noisier environment, the performance decreases to an F_1_-score of 0.931. Despite this degradation, the model continues to demonstrate reliable fault detection and localization capabilities.

The results confirm that the proposed framework is resilient to measurement noise and maintains high diagnostic accuracy even under degraded signal conditions. The gradual performance degradation with decreasing SNR further indicates that the model behavior is consistent and physically meaningful. This robustness can be attributed to the use of time–frequency representations and multi-sensor data fusion, which enhance the separability of fault-related features.

Despite high diagnostic accuracy, additional analyses under non-ideal conditions such as sensor reduction, control delays, and measurement noise show a gradual and consistent performance degradation. This behavior confirms that the model does not rely on data memorization but rather learns meaningful features that generalize effectively under varying conditions.

### Real-time performance evaluation

To evaluate the real-time applicability of the proposed open-circuit fault diagnostic framework, a quantitative latency analysis was conducted for the complete online processing chain, including signal acquisition, CWT preprocessing, data grouping, and ResNet-18 inference. The system processes 25,000 samples per diagnostic cycle at a sampling frequency of 10 MHz, resulting in an acquisition time of 2.5 ms .For each sensor signal, the average preprocessing latency comprising host-to-device transfer, CWT computation, resizing, and device-to-host transfer was measured on the Quadro RTX 3000 GPU-based MATLAB implementation as 20.68 ms per signal. Since six sensor channels are processed sequentially for each diagnostic instance, the total preprocessing latency is 124.1 ms. The average grouping time required to concatenate the six processed scalograms into a 118 × 500 × 6 input is 0.54 ms. In addition, the measured ResNet-18 inference latency is 9.66 ms per sample. Therefore, the total end-to-end diagnosis latency is 136.8 ms.

The latency analysis reveals that the dominant computational burden lies in the CWT preprocessing stage, which accounts for approximately 90.7% of the total execution time. In contrast, the CNN inference stage contributes only 7.1%, while acquisition and grouping overheads remain negligible. This indicates that the proposed framework is constrained by preprocessing and not by inference. Although the measured latency of 136.8 ms is acceptable for open-circuit fault diagnosis applications^9^, where fault identification is required within monitoring and maintenance timescales rather than microsecond-scale protection intervals, it also highlights that optimization of the wavelet preprocessing stage is the primary pathway for improving real-time performance.

Recent advances in fast Continuous Wavelet Transform (fCWT) have demonstrated acceleration factors ranging from 34 to 122 times over conventional CWT implementations while maintaining accuracy in both the time and frequency domains. Therefore, replacing the standard MATLAB CWT implementation with optimized fCWT can significantly reduce preprocessing latency^37^. In addition, FPGA-based implementations are highly feasible due to the inherent parallelism available for both wavelet transform and convolutional neural network acceleration^38^. Consequently, an FPGA implementation of the proposed framework is expected to reduce the total latency substantially below the current MATLAB GPU implementation, potentially enabling real-time diagnostic performance suitable for industrial applications. These results confirm the practical feasibility of applying the proposed framework in real-time industrial fault diagnosis systems.

## Conclusion

This work presented a comprehensive multi-label fault localization framework for a three-port DAB converter, integrating time–frequency analysis with deep learning. Midpoint voltage measurements $\:({\mathrm{V}}_{\mathrm{A}}-{\mathrm{V}}_{\mathrm{F}})$ were transformed into CWT scalogram representations to extract strong transient and spectral features associated with open-circuit fault events.

A ResNet-18 architecture was developed using sigmoid activation and binary cross-entropy loss to enable simultaneous identification of multiple faults. The proposed model achieved excellent diagnostic performance on unseen test data, with both micro- and macro-averaged F₁-scores of 0.9942, confirming its ability to accurately discriminate fault patterns across all converter switches.

Robust analyses demonstrated the adaptability of the framework under several challenging operating scenarios. When tested on unseen three-switch simultaneous faults, despite being trained only on single- and two- switch fault classes, the model maintained high generalization capability with a micro-F₁ of 0.8464. Under realistic dead-time variations ranging from 1 µs to 5 µs, the framework maintained reliable fault detection performance, with F₁-scores ranging from 0.952 to 0.961, demonstrating robustness against practical timing distortions in converter operation. Measurement noise analysis showed that the framework remains resilient under realistic noisy environments, maintaining F₁-scores of 0.979 at 40 dB SNR and 0.931 at 30 dB SNR. In addition, sensor reduction tests further showed that high diagnostic accuracy can be retained even when two-thirds of the voltage sensors are removed, confirming the practicality of the method for hardware-constrained implementations.

A comparison with an equivalent multi-class ResNet-18 classifier revealed that the proposed multi-label approach more effectively captures overlapping fault conditions and simultaneous switching failures, making it better suited for multi-port converters where simultaneous faults naturally occur.

Real-time performance evaluation further confirmed the practical feasibility of the proposed framework. The complete end-to-end diagnosis latency, including signal acquisition, CWT preprocessing, data grouping, and CNN inference, was measured as 136.8 ms in the current MATLAB GPU implementation. The analysis shows that the dominant computational burden arises from the CWT preprocessing stage, while the neural network inference contributes only a small fraction of the total latency. These results indicate that the proposed framework is suitable for open-circuit fault diagnosis applications and can be further accelerated through optimized fast CWT algorithms or FPGA-based implementations for deployment in practical industrial converter monitoring systems.

Future work will focus on experimental validation using hardware prototypes and extending the framework to additional fault types, including short-circuit and sensor faults, and component degradation effects. In addition, real-time implementation will be explored through optimized signal processing techniques and FPGA-based acceleration. The integration of the proposed model with online fault mitigation strategies will also be investigated to enhance system reliability in practical applications.

## Data Availability

All data generated or analyzed during this study are included in this published article.
